# The Osteogenic Properties of Calcium Phosphate Cement Doped with Synthetic Materials: A Structured Narrative Review of Preclinical Evidence

**DOI:** 10.3390/ijms24087161

**Published:** 2023-04-12

**Authors:** Siti Sarah Md Dali, Sok Kuan Wong, Kok-Yong Chin, Fairus Ahmad

**Affiliations:** 1 Department of Pharmacology, Faculty of Medicine, Universiti Kebangsaan Malaysia, Jalan Yaacob Latif, Bandar Tun Razak, Cheras, Kuala Lumpur 56000, Malaysia; aramddali96@gmail.com (S.S.M.D.); chinkokyong@ppukm.ukm.edu.my (K.-Y.C.); 2 Department of Anatomy, Faculty of Medicine, Universiti Kebangsaan Malaysia, Jalan Yaacob Latif, Bandar Tun Razak, Cheras, Kuala Lumpur 56000, Malaysia; fairusahmad@ukm.edu.my

**Keywords:** biomimetic materials, bone defect, chemical elements, polymers

## Abstract

Bone grafting is commonly used as a treatment to repair bone defects. However, its use is challenged by the presence of medical conditions that weaken the bone, like osteoporosis. Calcium phosphate cement (CPC) is used to restore bone defects, and it is commonly available as a bioabsorbable cement paste. However, its use in clinical settings is limited by inadequate mechanical strength, inferior anti-washout characteristics, and poor osteogenic activity. There have been attempts to overcome these shortcomings by adding various natural or synthetic materials as enhancers to CPC. This review summarises the current evidence on the physical, mechanical, and biological properties of CPC after doping with synthetic materials. The incorporation of CPC with polymers, biomimetic materials, chemical elements/compounds, and combination with two or more synthetic materials showed improvement in biocompatibility, bioactivity, anti-washout properties, and mechanical strength. However, the mechanical property of CPC doped with trimethyl chitosan or strontium was decreased. In conclusion, doping of synthetic materials enhances the osteogenic features of pure CPC. The positive findings from in vitro and in vivo studies await further validation on the efficacy of these reinforced CPC composites in clinical settings.

## 1. Introduction

Bone grafts are used to fill missing bone segments, improve skeletal projection, and provide mechanical support in bone defects by promoting osseous ingrowth, providing a structural substrate, and acting as a vehicle for controlled drug delivery in bone healing [1,2]. Although bone grafting is a widely utilised treatment to rebuild bone, the management of bone defects remains a great challenge, especially in individuals with medical conditions which compromise the bone healing process, such as osteoporosis, diabetes, and hypothyroidism [3]. An excellent bone graft should meet the characteristics of bioactive, biocompatible, osteoinductive, osteoconductive, resorbable, and high mechanical strength [4].

Autogenous bone graft represents the gold standard for treating bone defects because it does not cause immunoreaction and has osteoconductive properties. However, the use of autogenous bone grafts in clinical practice is restricted by limited bone grafts that are readily available, as well as the high incidence of complications at the donor and recipient sites [5]. Other alternatives used to optimise treatment include allografts and xenografts. Donor site morbidity issues are avoided by using these alternatives. However, they require sterilisation and purification, do not produce osteoconductive signals, and lack living cells. Additionally, they have the potential to cause bacterial or viral infections as well as a host tissue immunological reaction after implantation [6]. Herein, artificially produced bone grafts have been considered for the reasons of unlimited supply, minimal risk of disease transmission or immunoreaction, easy sterilisation and storage, as well as the availability of different shapes and sizes for surgical applications [7]. 

Calcium phosphate cement (CPC) is a synthetic self-setting material serving as an alternative to treat bone defects. CPC comprises a liquid phase and a calcium phosphate-solid phase in which they react chemically to form hydroxyapatite when combined. CPC offers the advantages of biocompatibility, injectability, mouldability, and hardening in situ, allowing optimal bone tissue-implant contact even in irregular defect dimensions and minimally invasive surgery, thus making it a highly appealing bone substitute in surgical applications [8]. However, the major drawbacks of CPC are brittleness, low mechanical strength, inferior anti-washout, and the lack of osteogenic ability, which decreases the implant stability that limits its application to non-stress bearing locations. To overcome these shortcomings, many studies have been conducted to design and fabricate CPCs enhanced by biological and synthetic materials with superior mechanical strength and osteogenic properties. 

**Figure 1:**
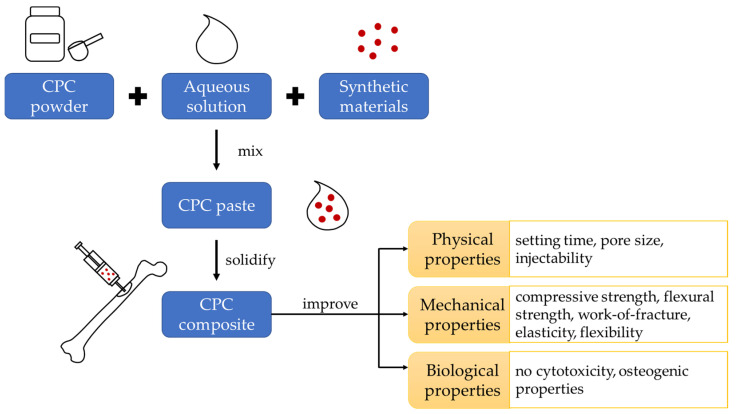
Conceptual framework of the review.

A recent review has been published to summarise the incorporation of CPC with materials derived from living organisms (including bone-related transcription factors, proteins, polysaccharides, and blood components) in treating bone defects [9]. Herein, the current review confers a comprehensive overview of the CPC reinforced by synthetic materials on their physical, mechanical, and biological properties (Figure 1). The available evidence indicates the strategies are reinforcement with synthetic polymers, biomimetic materials, chemical elements or compounds, and the combination of several synthetic materials.

## 2. Literature Search

**Figure 2:**
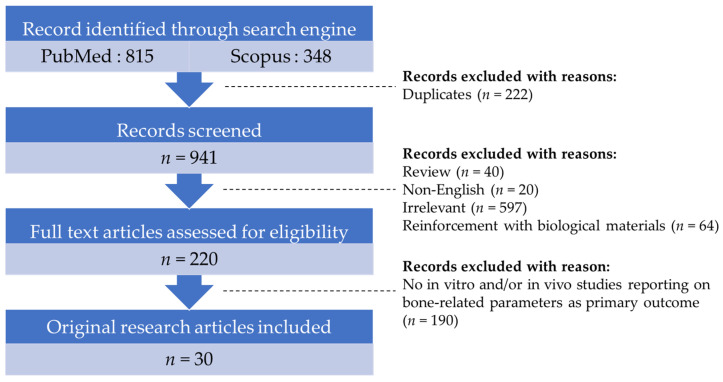
Evidence collection framework.

Literature acquisition was performed using the PubMed and Scopus databases with the search string: (enhancement OR improvement OR reinforcement) AND (calcium phosphate cement) AND (bone OR osteoporosis OR fracture OR osteoblast OR osteoclast OR osteocyte). From the search, we obtained 815 and 348 records from inception until 15 January 2023 from PubMed and Scopus, respectively. Duplicate articles (*n* = 222) were excluded. The titles and abstracts were initially screened to exclude reviews, non-English, articles, books, book chapters, commentaries, conference papers, letters to the editor, meta-analyses, and irrelevant articles. Subsequently, the full-text articles were screened based on the inclusion and exclusion criteria. The main objective of this review is to summarise the characteristics of CPC enhanced with synthetic materials, defined as materials made by humans through chemical synthesis. The exclusion criteria of this review are (a) the reinforcement of CPC with biological materials derived from living organisms; (b) original research articles not reporting bone parameters as the primary outcomes; and (c) original research articles with the absence of in vitro and in vivo experimental methods. A total of 30 relevant original research articles were included in this review. The evidence collection framework is summarised in Figure 2.

## 3. The Enhancement of CPC Using Synthetic Materials

### 3.1. Synthetic Polymers

Synthetic polymers refer to macromolecules artificially produced in laboratories through repetitive bonding of multiple monomers [10,11]. Several polymers, including poly(lactic-co-glycolic acid) (PLGA), PEGylated poly(glycerol sebacate) (PEGS), and sodium polyacrylate (PAAS), have been incorporated into CPC to enhance its properties (Table 1). 

PLGA is produced by the co-polymerisation of glycolic acid and lactic acid. It was commonly used as an enhancer for CPC as it has excellent biocompatibility, and the degradation time can be controlled. A study by Lu et al. showed that the addition of PLGA with different particle morphologies influenced the characteristics of CPC. Their findings indicated that CPC incorporated with dense spherical and irregularly shaped PLGA had proper setting time and improved compressive strength, whereas porous PLGA prolonged final setting time and decreased compressive strength of CPC. However, PLGA favoured cell proliferation of mouse bone mesenchymal stem cells regardless of their morphologies [12]. In another study, the addition of PLGA fibres electrospun on CPC exhibited higher flexural strength and work-of-fracture as compared to the CPC without PLGA fibres. The seeding of human umbilical cord mesenchymal stem cells (hUCMSCs) on CPC incorporated with PLGA fibres showed rapid cell proliferation and mineralisation. The expressions of alkaline phosphatase (ALP), osteocalcin (OCN), and collagen type I (COL1) were higher in hUCMSCs cultured on CPC containing PLGA [13]. Likewise, good cell proliferation and growing ALP activity were detected using rat bone marrow mesenchymal stem cells (rMSCs) seeded on PLGA/CPC scaffolds [14]. In the same study, the implantation of CPC with PLGA on bone defects at the femora of New Zealand white rabbits showed that the implant was gradually replaced by the host’s new bones and numerous osteoblasts after 16 weeks [14]. A study done by Maenz et al. also showed improved bone microstructure, bone mineral density (BMD), bone biomechanical compression strength, and static bone histomorphometry in ageing osteopenic female sheep with lumbar vertebrae bone defects and subjected to implantation using CPC incorporated with PLGA after 3 and 9 months [15]. 

PEGS is a biodegradable elastomer formed from the polycondensation of glycerol and sebacic acid. It has been developed to address the drawbacks of CPC in enhancing mechanical robustness, biocompatibility, bioactivity, and osteogenic activity for bone regeneration [16,17]. Ma et al. conducted in vitro and in vivo experiments to investigate the effects of PEGS-modified CPC scaffold for bone regeneration using rMSCs and rat calvarial defect model. The results indicated an increase in cell viability, cell proliferation, cell attachment, and osteogenic differentiation of rMSCs on PEGS/CPC scaffolds. New bone formation, greater mineralisation rate, higher bone volume/total volume (BV/TV), and promoted osteogenesis were also detected in rats subjected to two critical-sized (5 mm) calvarial defects and implanted with PEGS/CPC scaffold [16]. 

PAAS is a cross-linked polymer containing sodium with a super-absorbing ability. It is non-toxic and biocompatible, suggesting that it is frequently used as a food additive and has a potential application in drug delivery. When dissolved in aqueous solutions, PAAS has a high viscosity, strong hydrophilicity, and shape retention properties, thus rendering it a potential enhancer to enhance the anti-washout property of CPC [18]. A previous study demonstrated that the incorporation of PAAS into CPC enhanced the anti-washout, injectability, and compressive strength of the cement paste while retaining the setting time and material microstructure. The mouse mesenchymal stem cells were well-adhered, spread, and proliferated when incubated on PAAS/CPC in vitro [18].

**Table 1:** Bone-sparing properties of CPC enhanced by polymers.

Enhancer	Type of Study	Cell Culture/Animal Model	Findings	References
Dense & irregularly shaped PLGA	In vitro	Mouse bone mesenchymal stem cell	Cell proliferation: ↑, setting time: ↔, degradation rate: ↑, compressive strength: ↑, good biocompatibility	[12]
Porous PLGA			Cell proliferation: ↑, final setting time: ↑, compressive strength: ↓, good biocompatibility	
PLGA	In vitro	hUCMSCs	Flexural strength: ↑, work-of-fracture: ↑, cell proliferation: ↑, ALP: ↑, OCN: ↑, COL1: ↑, mineralisation: ↑	[13]
PLGA	In vitro	rMSCs	Bone regeneration: ↑	[14]
	In vivo	Defect in the femora of New Zealand white rabbits	Cell proliferation: ↑, ALP activity: ↑	
PLGA	In vivo	Bone defect at L4 lumbar vertebral body in aged osteopenicfemale sheep	BV/TV: ↑, BMD: ↑, biomechanical compression strength: ↑, bone erosion: ↓, osteoid volume: ↑, osteoid surface: ↑	[15]
PEGS	In vitro	rMSCs	Cell viability: ↑, cell proliferation: ↑, cell attachment: ↑, COL1: ↑, Runx-2: ↑, OCN: ↑	[16]
	In vivo	Calvarial defect model of male Sprague- Dawley rats	BV/TV: ↑, Tb.Th: ↑, mineralisation: ↑	
PAAS	In vitro	Mouse bone marrow stromal cells	Setting time: ↑, compressive strength: ↑, cell proliferation: ↑	[18]

Abbreviations: ALP, alkaline phosphatase; BV/TV, bone volume/total volume; COL1, collagen type I; hUCMSC, human umbilical cord mesenchymal stem cell; PAAS, sodium polyacrylate; PEGS, PEGylated poly (glycerol sebacate); PLGA, poly(lactic-co-glycolic acid); rMSCs, rat bone marrow mesenchymal stem cells; Tb.Th, trabecular thickness; OCN, osteocalcin; ↑, increase; ↓, decrease; ↔, no change.

### 3.2. Biomimetic Materials

Biomimetic materials are known as artificial synthetic materials which imitate biological substances of living organisms [19]. A variety of biological signalling cues are required to provide an optimal environment for the physiological bone healing process. Thus, biomimetic materials can be alternatives to replicate the configuration of the microenvironment in natural bone tissue. Synthetic collagen I mimetic P-15, trimethyl chitosan, and chondroitin sulphate are examples of biomimetic materials incorporated into CPC (Table 2). 

Synthetic collagen I mimetic P-15 is a synthetic 15-amino-acid sequence that is identical to the alpha I chain of COL1. It can bind with an inorganic bone matrix (hydroxyapatite), creating ideal conditions of biocompatibility, biodegradability and osteoconduction [20]. Since both CPC and P-15 have similar beneficial properties of osteoinductivity, osteoconductivity, and resorbability, their combination offers synergistic features in maintaining the stability of composite compared to the primary material [21]. A study using human mesenchymal stem cells seeded on chamber slides coated with CPC containing synthetic collagen I mimetic P-15 showed that the osteogenic differentiation was increased, evidenced by higher ALP, osteopontin (OPN), Runt-related transcription factor 2 (Runx-2), COL1, osteonectin, and OCN [21]. In addition, calcium deposits were detected in cells cultured on CPC with synthetic collagen I mimetic P-15 as an enhancer. Using the vertebrae of non-osteoporotic and osteoporotic sheep, the same group of researchers found that the bone augmented with CPC containing synthetic collagen I mimetic P-15 exhibited greater pull-out strength after pedicle screw insertion [21]. 

Trimethyl chitosan is a quarternised hydrophilic derivative of chitosan, which outperformed the parent molecule by its superior water solubility, biodegradable, biocompatible, and bioadhesive [22]. As it solves the well-known solubility drawback of chitosan, trimethyl chitosan is used as a reinforcing agent added to liquid CPC. The trimethyl chitosan-modified CPC had a longer setting time, improved wettability, and increased load-bearing capacity while maintaining the elasticity and bending strength compared to CPC without trimethyl chitosan additive. In vitro, the osteoblastic-like (MG-63) cells showed increased cell viability on CPC added with trimethyl chitosan, indicating good biocompatibility of the materials [23]. 

Chondroitin sulphate is a predominant glycosaminoglycan made up of alternating glucuronic acid and N-acetylgalactosamine disaccharide units [24]. It is an important structural component of the extracellular matrix network in bone and cartilage, incorporating fibronectin and growth factors to facilitate cell adhesion, migration, proliferation, and differentiation. Chondroitin sulphate can be animal-derived or manufactured synthetically. It has been introduced into CPC to enhance its osteogenic and integration abilities. Shi et al. (2019) reported that the setting time was prolonged, injectability was improved, and the fibronectin adsorption amount favouring cell adhesion was increased in the CPC paste reinforced with chondroitin sulphate relative to CPC per se. The bone mesenchymal stem cells also showed higher cell proliferation, cell differentiation, as well as osteogenic expression of ALP and OPN when cultured on the chondroitin sulphate-added CPC [25].

**Table 2:** Bone-sparing properties of CPC enhanced by biomimetic materials.

Enhancer	Type of Study	Cell Culture/Animal Model	Findings	References
Synthetic collagen I mimetic P-15	In vitro	Human mesenchymal stem cell	ALP: ↑, OPN: ↑, Runx-2: ↑, COL1: ↑, osteonectin: ↑, OCN: ↑, calcium deposit: ↑	[21]
	In vivo	Sheep vertebra of non- osteoporotic and osteoporotic model	Pull-out strength: ↑	
Trimethyl chitosan	In vitro	MG-63 cells	Setting time: ↑, specific surface area: ↓, wettability: ↑, compressive strength: ↔, elastic modulus: ↑, bending strength: ↑, cell viability: ↑, biocompatibility: ↑, load-bearing capacity: ↑	[23]
Chondroitin sulphate	In vitro	Bone mesenchymal stem cells	Setting time: ↑, injectability: ↑, fibronectin adsorption: ↑, cell proliferation: ↑, Runx-2: ↑, COL1: ↑, OCN: ↑, ALP: ↑, OPN: ↑	[25]

Abbreviations: ALP, alkaline phosphatase; BMD, bone mineral density; BV/TV, bone volume/total volume; COL1, collagen type I; OCN, osteocalcin; OPN, osteopontin; Runx-2, Runt-related transcription factor 2; ↑, increase; ↓, decrease; ↔, no change.

### 3.3. Chemical Elements and Compounds

Chemical elements are defined as substances that cannot be decomposed into simpler materials by the normal chemical process. On the other hand, chemical compounds refer to substances that contain two or more chemical elements held together by chemical bonds. Both chemical elements and compounds, such as strontium, selenium, iron, zinc, copper, magnesium, lithium, silicon, and calcium silicate, have been incorporated into CPC as enhancers (Table 3). 

Similar to calcium, strontium is located in group 2 of the periodic table, indicating the comparable chemical properties and biological functions between these elements. Strontium exists as a trace element in the human body, and it concentrates in the bones [26]. With the distinct ability to intensify osteoblastogenesis and suppress osteoclastogenesis, strontium-releasing CPC might be a promising material for the regeneration of bone defects. The reinforcement of CPC by strontium resulted in no significant difference in the setting time but greater compressive modulus in comparison to CPC alone [27,28]. Two in vitro studies demonstrated higher cell proliferation and ALP activity in MG-63 cells [28] and primary human mesenchymal stromal cells seeded on strontium-doped CPC [27]. The performance of strontium-loaded CPC on defect at the non-load bearing (distal femoral condyle) and load-bearing sites (proximal tibia metaphysis) of female merino sheep were evaluated. After 26 weeks of implantation, the bone area and proportion of materials covered with bone were increased, but material degradation and osteoclast formation were not affected [29]. The clinical applicability of strontium-modified CPC in balloon kyphoplasty was tested in an 80-year-old male cadaver via vertebral body reconstruction, in which the sample was injected into the vertebral bodies of a human cadaver. The findings revealed that the strontium-containing CPC had higher viscosity, thus a lower tendency to leak out into the surrounding tissue during treatment compared to the control, PMMA cement [27]. In another study, male Sprague-Dawley rats with calvarial defect and implanted with nanostrontium-loaded CPC had increased bone formation, as well as higher expressions of bone morphogenetic protein-2 (BMP-2), OCN, and osteoprotegerin (OPG) in comparison to those implanted with CPC without nanostrontium [30]. Strontium ranelate is one of the anti-osteoporosis medications in postmenopausal women. The addition of strontium ranelate into CPC caused an increase in cell spreading area, cell proliferation, and expression of COLI, ALP, OCN, and Runx-2 after the culture of mouse bone marrow mesenchymal stem cells [31]. The expression of osteoclastogenesis-related genes coding for tartrate-resistant acid phosphatase (TRAP), cathepsin K (CTSK), matrix metalloproteinase 9 (MMP-9), and carbonic anhydrase II (Car2) was downregulated in the murine macrophage (RAW264.7) cells cultured on strontium ranelate-containing CPC [31]. Although the combination of strontium ranelate and CPC is a promising formula to stimulate osteogenesis and new bone formation, the potential cardiovascular risks of strontium ranelate should not be neglected. 

Selenium is an essential trace mineral required for the synthesis of selenoproteins. The skeletal-promoting effects of selenium have been widely established in vivo, whereby selenium deficiency was associated with impaired bone health which can be reversed through selenium supplementation [32]. Given the potential beneficial effects of selenium on bone, selenium-based biomaterials have been developed to promote bone tissue regeneration. An ovariectomised rat model with bone defect at the femoral epiphysis was utilised to study the effects of selenium in enhancing the efficacy of CPC in the treatment of osteoporotic bone defect for 12 weeks. Micro-computed tomography analysis pointed out that the animals treated with selenium-added CPC exhibited higher BMD, BV/TV, trabecular number (Tb.N), connectivity density (Conn.D), trabecular thickness (Tb.Th), but lower trabecular separation (Tb.Sp). The implantation of selenium-added CPC also resulted in greater new bone formation, mineral apposition rate (MAR), and biomaterial biodegradation at the defect site. The levels of superoxide dismutase (SOD), glutathione peroxidase (GPX), and OPG were raised, whereas the expression of catalase (CAT) and receptor activator of nuclear factor-kappa B ligand (RANKL) were decreased. These findings indicated that the rapid bone-repairing ability of this material might be in part achieved through suppression of oxidative stress and receptor activator of nuclear factor-kappa B (RANK)/RANKL/OPG pathway [33]. 

Iron is a mineral naturally present in many types of foods and available as a dietary supplement. It is a necessary component of haemoglobins, enzymes, and cytochromes. Accumulating evidence suggested that iron deficiency exerted a negative impact on bone. Female rats fed on an iron-restricted diet had compromised trabecular bone microstructure at lumbar vertebrae [34,35]. Hence, iron sufficiency plays an important role in bone regeneration. Zhang et al. reported that the setting time was shortened, but the injectability and compressive strength were increased in the iron-doped CPC relative to CPC alone. The effects of these materials on osteogenesis and angiogenesis were tested using two different cells, mouse bone marrow stromal cells and human umbilical vein endothelial cells (HUVECs). The cell proliferation of mouse bone marrow stromal cells and expression of ALP, COL1, OPN, and Runx-2 were elevated when incubated with iron-modified CPC. Higher levels of vascular endothelial growth factor (VEGF) and endothelial nitric oxide synthase (eNOS) were also detected in HUVECs when cultured on CPC containing iron [36]. Moreover, iron-incorporated CPC demonstrated good biocompatibility with new bone formation as well as no sign of inflammation and necrosis at the defect site in female sheep after implantation [37]. 

Zinc is a crucial mineral for the growth, development, and maintenance of healthy bones [38]. A meta-analysis conducted by Ceylan et al. pinpointed that serum zinc concentration was lower in osteoporotic patients than in healthy subjects, and zinc supplementation was effective in increasing bone formation markers and BMD [39]. Based on the beneficial role of zinc in maintaining normal physiological bone growth, the use of zinc as an additive to reinforce CPC for bone tissue regeneration is hypothesised. Two in vitro studies found higher cell viability, cell proliferation, ALP activity, and osteogenic gene expression in murine mesenchymal stem cells and MC3T3-E1 cells seeded on zinc-modified CPC as compared to the cement without zinc [40,41]. 

Magnesium is the second most abundant intracellular cation in the human body after potassium. Approximately 60% of magnesium is present in bone. Magnesium deficiency negatively impacts bone health in different ways: (a) it directly increases osteoclastic activity and decreases osteoblastic activity; (b) it indirectly induces hormonal changes (such as parathyroid hormone and vitamin D) as well as promotes inflammatory response and oxidative stress, leading to bone loss [42]. Apart from its physiological role in maintaining musculoskeletal health, magnesium is a degradable metal with potential use as an alternative for non-resorbable materials in the fabrication of implantable medical devices [43]. The bone cell response to CPC doped with magnesium has been investigated in vitro and in vivo by two groups of investigators. Zhang et al. revealed higher fibronectin adsorption, cell attachment, integrin α5β1, ALP activity, COL1 and OCN expression in bone marrow stromal cells cultured on CPC reinforced with magnesium than pure CPC [44]. Another study also showed higher cell proliferation of MG-63 cells cultured on CPC mixed with magnesium [45]. Besides, both studies showed increased osteogenesis and new bone formation in in vivo studies using the calvarial bone defect model in rats and rabbits. 

Copper is a vital trace element in the human body for the proper functioning of organs and metabolic processes. Adequate serum copper level is important in maintaining good bone health, whereby lower concentration was associated with decreased BMD, and higher concentration was associated with increased fracture risk [46]. Considering the potential bone-protecting effects of copper, its application can be broadened to the repairing of bone defects. With the addition of copper, there were increases in setting time, compressive strength, and injectability of the CPC. However, there was no significant difference in the porosity of the CPC after the addition of copper. Mouse bone marrow mesenchymal stem cells seeded on CPC containing copper displayed higher adhesion activity, cell proliferation, and osteogenic expression of COL1, OCN, and ALP. HUVECs cultured on the combination of CPC and copper ions showed higher expression of angiogenesis-related genes [including eNOS, VEGF, basic fibroblast growth factor (bFGF), and nitric oxide] compared to pure CPC [47]. 

Apart from its use in the treatment of bipolar disorder, lithium has received much attention for its osteoprotective properties [48]. Lithium is a well-known inhibitor of glycogen synthase kinase-3 beta (GSK3β), a protein kinase that modulates the canonical Wingless (Wnt)/beta (β)-catenin pathways via phosphorylation of β-catenin as the downstream target [49]. Hence, lithium can be an excellent candidate to be incorporated into CPC to enhance bone regeneration. A lithium chloride-doped CPC was developed and tested for bone regenerative effects in MC3T3-E1 cells and ovariectomised rats with bone defects. The in vitro experiment showed higher cell proliferation, cell differentiation, mineralisation, and osteogenic differentiation after the incubation of MC3T3-E1 cells on a lithium/CPC scaffold. Mechanistically, the osteogenic properties of lithium-modified CPC were mediated through activation of the Wnt/β-catenin pathway, indicated by a higher level of phosphorylated GSK3β and a lower level of phosphorylated β-catenin. In in vivo study, lithium-modified CPC was implanted on bone defect created at the medial tibial shaft of female Sprague-Dawley rats. The findings showed higher BV/TV and increased new bone formation around the defect site implanted with lithium and CPC [50]. 

Silicon carbide whiskers are fibre-like materials produced by mixing and sintering silicon carbide fibres and alumina powder. It has excellent elasticity, strength, hardness, and chemical stability (such as wear, corrosion, and temperature resistance), thus, is commonly used as a reinforcement material for ceramics, metals, and plastics for a wide range of industrial applications [51]. With these features, silicon carbide whiskers were fused into CPC to overcome its brittleness, resulting in superior strength and toughness for weight-bearing applications. An early study found that the compressive strength, flexural strength, and elastic modulus of CPC were elevated after the addition of silicon carbide whiskers. However, the MC3T3-E1 cells cultured on pure CPC and silicon carbide whiskers/CPC composite showed similar live cell density, cell adhesion, cell viability, and cell proliferation [52]. 

Calcium silicate is a compound synthesised by reacting calcium oxide and silica at different ratios [53]. Both calcium and silica have a wide application for bone tissue engineering, mainly attributed to their role in promoting osteogenic differentiation and bone calcification, respectively [54]. The outstanding bioactivity and biocompatibility of calcium silicate make it a promising bioceramic in the field of dentistry and orthopaedics [53]. Zhao and colleagues proved that the MC3T3-E1 cells and HUVECs cultured on CPC incorporated with calcium silicate had higher cell proliferation and ALP activity [55].

**Table 3:** Bone-sparing properties of CPC enhanced by chemical elements/compounds.

Enhancer	Type of Study	Cell Culture/Animal Model	Findings	References
Strontium	In vitro	MG-63 cells	Setting time: ↓, compressive strength: ↔, cell proliferation: ↑, ALP activity: ↑	[28]
Strontium	In vitro	Primary human mesenchymal stromal cells	Setting time: ↔, compressive strength: ↑, cell proliferation: ↑, ALP activity: ↑,	[27]
	In vivo	Vertebral body reconstruction in an 80-year-old male cadaver	Viscosity: ↑, tendency to leak out: ↓	
Strontium	In vivo	Calvarial bone defect in male Sprague-Dawley rats	Bone formation: ↑, BMP-2: ↑, OCN: ↑, OPG: ↑	[30]
Strontium	In vivo	Two bone defects, one at distal femoral condyle and one at proximal tibial metaphysis of adult female merino sheep	B.Ar/T.Ar: ↑, material resorption: ↔, osteoclast number: ↔	[29]
Strontium ranelate	In vitro	Mouse bone marrow mesenchymal stem cells	Cell spreading area: ↑, cell proliferation: ↑, COL1: ↑, ALP: ↑, OCN: ↑, Runx-2: ↑	[31]
	In vitro	RAW264.7 cells	TRAP: ↓, CTSK: ↓, MMP-9: ↓, Car2: ↓	
Selenium	In vivo	Bone defect at femoral epiphysis ofovariectomised rats	BMD: ↑, BV/TV: ↑, Tb.N: ↑, Conn.D: ↑, Tb.Th: ↑, Tb.Sp: ↓, bone regeneration: ↑, biomaterial degradation: ↑, MAR: ↑, SOD: ↑, CAT: ↓, GPX: ↑, OPG: ↑, RANKL: ↓	[33]
Iron	In vitro	Mouse bone marrow stromal cells	Setting time: ↓, injectability: ↑, compressive strength: ↑, cell proliferation: ↑, ALP: ↑, COL1: ↑, OPN: ↑, Runx-2: ↑	[36]
		Human umbilical vein endothelial cells	VEGF: ↑, eNOS: ↑	
Iron	In vivo	Four bone defects created at the proximal and distal extremities of the humerus and femur in female Romanian alpine sheep	New bone and blood vessel formation: ↑, cells at resorption zone: ↑, osteoid formation: ↑, no inflammation & necrosis	[37]
Zinc	In vitro	Mouse bone mesenchymal stem cells	Cell proliferation: ↑, ALP activity: ↑, COL1: ↑, Runx-2: ↑	[40]
Zinc	In vitro	MC3T3-E1 cells	Setting time: ↓, tensile strength: ↑, cell viability: ↑, cell proliferation: ↑, ALP activity: ↑	[41]
Magnesium	In vitro	Rat bone marrow stromal cells	Fibronectin adsorption: ↑, cell attachment: ↑, integrin α5β1 expression: ↑, ALP: ↑, COL1: ↑, OCN: ↑	[44]
	In vivo	Calvarial defect of 4-month-old Sprague Dawley rats	New bone formation: ↑, material residue: ↓	
Magnesium	In vitro	MG-63 cells	Cell proliferation: ↑	[45]
	In vivo	Calvarial defect in New Zealand rabbits	New bone formation: ↑	
Copper	In vitro	Mouse bone marrow mesenchymal stem cells	Setting time: ↑, compressive strength: ↑, porosity: ↔, injectability: ↑, adhesion activity: ↑, cell proliferation: ↑, COL1: ↑, OCN: ↑, ALP: ↑	[47]
		Human umbilical vein endothelial cells	eNOS: ↑, VEGF: ↑, bFGF: ↑, nitric oxide: ↑	
Lithium chloride	In vitro	MC3T3-E1 cells	Cell proliferation: ↑, ALP activity: ↑, mineralisation: ↑, COL1: ↑, OCN: ↑, OPG: ↑, Runx-2: ↑, p-β-catenin: ↓, p-GSK3β: ↑	[50]
	In vivo	Bone defect at the medial tibial shaft of female ovariectomised Sprague-Dawley rats	BMD: ↑, new bone formation: ↑, the gap was occupied by new bone.	
Silicon carbide whiskers	In vitro	MC3T3-E1 cells	Flexural strength: ↑, work-of-fracture: ↑, elastic modulus: ↑, hardness: ↑, cell adhesion: ↔, cell viability: ↔, cell proliferation: ↔	[52]
Calcium silicate	In vitro	MC3T3-E1 cells	Cell proliferation: ↑, ALP activity: ↑	[55]
		Human umbilical vein endothelial cell		

Abbreviations: ALP, alkaline phosphatase; B.Ar/T.Ar, bone area/total area; BMD, bone mineral density; BMP-2, bone morphogenetic protein-2; BV/TV, bone volume/total volume; Car2, carbonic anhydrase II; CAT, catalase; COL1, collagen type I; Conn.D, connectivity density; CTSK, cathepsin K; eNOS, endothelial nitric oxide synthase; GPX, glutathione peroxidase; MAR, mineral apposition rate; MMP-9, matrix metalloproteinase 9; OCN, osteocalcin; OPG, osteoprotegerin; OPN, osteopontin; p-GSK3β, phosphorylated glycogen synthase kinase-3 beta; RANKL: receptor activator of nuclear factor-kappa B ligand; Runx-2, Runt-related transcription factor 2; SOD, superoxide dismutase; Tb.N, trabecular number; Tb.Sp, trabecular separation; Tb.Th, trabecular thickness; TRAP, tartrate-resistant acid phosphatase; VEGF, vascular endothelial growth factor; ↑, increase; ↓, decrease; ↔, no change.

### 3.4. Combination between Two or More Synthetic Materials

Two or more synthetic materials can be combined/mixed to enhance CPC (Table 4). PLGA has been incorporated into the CPC as an individual enhancer or in combination with other synthetic materials, such as wollastonite, perfluorocarbon, silicon/zinc, simvastatin/strontium, and alendronate.

Wollastonite is a naturally occurring mineral composed of calcium, silicon, and oxygen. It possesses outstanding performance in apatite mineralisation, biocompatibility, biodegradability, non-toxicity, mechanical properties, osteogenesis, vascularisation, and the ability to release bioactive silicon ions, suggesting its significant application in bone tissue regeneration [56]. Wollastonite has a rapid rate of decomposition, causing a rise in the pH level in the local environment and, subsequently, the release of excessive silicon ions, which can be harmful to cells. For this reason, wollastonite is not typically employed as a bone graft individually [57]. Qian et al. revealed that the flexibility was increased when wollastonite was mixed with PLGA as the enhancer for CPC. The cell attachment, cell proliferation, and expression of Runx-2, COL1, and BSP of mouse bone mesenchymal stem cells on wollastonite/PLGA/CPC composite were improved. Implanted material with wollastonite/PLGA/CPC on bone defect at the femoral condyle of New Zealand rabbits also showed increases in new bone formation, bone matrix, new blood vessels, and a decrease in material residual [57].

Perfluorocarbons are synthetic colourless, odourless, non-flammable, and unreactive compounds consisting of fluorine and carbon. It can dissolve oxygen, thus playing a crucial role in the delivery of oxygen for organ preservation [58]. Recent evidence reported the bone fracture healing properties of nanoscale perfluorocarbon in a rabbit model with radial fractures, as shown by the elevations in soft callus formation, collagen synthesis, as well as the expression of VEGF, MMP-9, and OCN [59]. Perfluoro-15-crown-5-ether (PFCE) is a commonly used perfluorocarbon, which is chemically and biologically inert, temperature and storage stable, as well as posing no infectious risk. In a study conducted by Mastrogiacomo et al., a novel composite was created by combining PFCE, PLGA, and gold nanoparticles, followed by incorporation into CPC. This composite was tested in vivo using a rat femoral condyle defect model and the increase in new bone formation was seen [60].

Various trace elements are present in the natural physiological extracellular environment to facilitate osteogenesis. Therefore, mimicking the bone microenvironment would require more than one trace element as the doping material. Silicon stimulates collagen synthesis and vascularisation, whereas zinc promotes bone growth and mineralisation. A study by Liang et al. showed increases in initial and final setting time, injectability, and compressive strength when PLGA microsphere and silicon/zinc dual elements were presented in CPC. The osteoimmunomodulatory effects of PLGA/silicon/zinc/CPC scaffold were proven to be superior to either one of the materials mixed with CPC, indicated by higher production of BMP-2 in the rMSCs. The immunomodulatory effects of the composites were tested using the RAW 264.7 macrophages. Better cell adhesion and spreading, lower pro-inflammatory cytokines [tumour necrosis factor-alpha (TNF-α) and interleukin-6 (IL-6)], higher anti-inflammatory mediators [interleukin-10 (IL-10) and transforming growth factor-1 beta (TGF-1β)], as well as raised vascular genes [VEGF and platelet-derived growth factor-BB (PDGF-BB)] were detected in the RAW 264.7 cells seeded on PLGA/silicon/zinc/CPC composite. The implantation of PLGA/silicon/zinc/CPC scaffold on bone defect at the femur of male Sprague-Dawley rats also resulted in higher new bone formation, bone ingrowth, and BV/TV with a lower residual material area as early as week 4 [61].

Simvastatin is a hypolipidemic medication that can promote bone regeneration. Statin exerts pleiotropic effects, which include hypocholesterolemic and bone protective actions via the inhibition of the mevalonate pathway by blocking 3-hydroxy-3-methylglutaryl coenzyme A (HMG-CoA) reductase, an enzyme catalysing the conversion of HMG-CoA to mevalonic acid. Subsequently, the downstream synthesis of cholesterol is suppressed, whereas the expression of bone morphogenetic protein-2 is promoted via inhibition of protein prenylation using isoprenoid intermediates as substrates, such as farnesyl pyrophosphate (FPP) and geranylgeranyl pyrophosphate (GGPP) [62]. The combination of PLGA microsphere, simvastatin, and strontium improved CPC features. For instance, (a) PLGA microsphere provides a good delivery system due to its biodegradation potential; (b) statin enhances the stability of PLGA microspheres; and (c) strontium affects the reactivity of CPC by inducing osteoblastic activity and suppressing osteoclastic activity [30]. The implantation of PLGA/simvastatin/strontium/CPC scaffold for 8 weeks into parietal bone defects in rabbits showed superior osteogenic activities and biocompatibility [30].

Alendronate is a bisphosphonate medication to treat osteoporosis. The anti-resorptive effects of alendronate are mediated through several mechanisms: (a) it directly prevents the development and recruitment of osteoclast progenitors and promotes apoptosis of osteoclasts [63]; (b) it interferes with the mevalonate pathway by inhibiting farnesyl pyrophosphate synthase enzyme and reduced prenylated protein, thus inducing osteoclast apoptosis [64]. A study by van Houdt et al. incorporated alendronate and PLGA into CPC. The findings showed that the increase in alendronate content gradually increased the initial and final setting time but decreased the compressive strength of the composite. The biological performance of the composite was evaluated in vivo using ovariectomised female Wistar rats subjected to femoral condyle bone defect. Micro-computed tomography analysis showed the implanted material was in contact with surrounding bone tissue. In addition, the animals implanted with alendronate and PLGA-loaded CPC displayed stimulated bone formation and raised bone density along with the reduction of material remnants from week 4 to week 12 [3].

Laponite^®^ is a synthetic nanoclay which has been described as a biocompatible disk-shaped silicate [65]. The particle size on Laponite^®^ is 1 nm in thickness and 25–30 nm in diameter, with a negative charge on the surface and a positive charge at the edge. Laponite^®^ displays non-cytotoxic and osteogenic effects on human bone marrow stromal cells. It also has wide application in the fabrication of composites to enhance mechanical properties [66]. On the other hand, dexamethasone is an anti-inflammatory and immunosuppressive medication used to treat inflammatory conditions. Utilising an in vivo ectopic bone formation model, the muscle of rats implanted with scaffolds containing dexamethasone and BMP-2 had higher bone formation than those implanted with scaffolds containing BMP-2 only, reiterating that the presence of dexamethasone enhanced the osteogenic capability of BMP-2, thus potentially reducing the required dosage of BMP-2 for clinical application [67]. Both Laponite^®^ and dexamethasone were used in combination and added into CPC to enhance its properties. Higher compressive strength and modulus but shorter setting time were noted in CPC encapsulated with dexamethasone-loaded Laponite^®^ nanoplates. When tested in vitro, the proliferation of MG-63 cells was improved after being cultured on dexamethasone/Laponite^®^ nanoplates/CPC composite [68]. Although the osteogenic capability of dexamethasone has been reported, the adverse effects of glucocorticoid in inducing bone loss should be under careful consideration for its suitability to be used as an enhancer for bone tissue engineering.

**Table 4:** Bone-sparing properties of CPC enhanced by the combination of two or more synthetic materials.

Enhancer	Type of Study	Cell Culture/Animal Model	Findings	References
PLGA + wollastonite	In vitro	Mouse bone mesenchymal stem cells	Flexibility: ↑, cell attachment: ↑, cell proliferation: ↑, Runx-2: ↑, COL1: ↑, BSP: ↑	[57]
	In vivo	Bone defect at femoral condyle of New Zealand rabbits	New bone formation: ↑, material residual: ↓, bone matrix: ↑, new blood vessel: ↑	
PLGA + PFCE + gold nanoparticles	In vivo	Bone defect at femoral condyle of male Wistar rats	New bone formation: ↑	[60]
PLGA + silicon/zinc	In vitro	rMSCs	Setting time: ↑, injectability: ↑, compressive strength: ↑, BMP-2: ↑	[61]
		RAW 264.7 cells	cell adhesion & spreading: ↑, TNF-α: ↓, IL-6: ↓, IL-10: ↑, TGF-1β: ↑, VEGF, PDGF-BB: ↑	
	In vivo	Bone defect at femur of male Sprague- Dawley rats	New bone formation: ↑, BV/TV: ↑, residual material: ↓	
PLGA microspheres + simvastatin + nanostrontium	In vivo	Parietal bone defect in male New Zealand white rabbits	New bone area: ↑, good biocompatibility	[30]
PLGA + alendronate	In vivo	Bone defect at both femoral condyles of ovariectomised female Wistar rats	Setting time: ↑, compressive strength: ↓, bone formation: ↑, BMD: ↑	[3]
Dexamethasone + Laponite^®^ nanoplates	In vitro	MG63 cells	Compressive strength: ↑, setting time: ↓, cell proliferation: ↑	[68]

Abbreviations: BMD, bone mineral density; BMP-2, bone morphogenetic protein-2; BSP, bone sialoprotein; BV/TV, bone volume/total volume; COL1, collagen type I; IL-6, interleukin-6; IL-10, interleukin-10; PDGF-BB, platelet-derived growth factor-BB; PFCE, perfluoro-15-crown-5-ether: PLGA, poly(lactic-co-glycolic acid); rMSCs, rat bone marrow mesenchymal stem cells; Runx-2, Runt-related transcription factor 2; TGF-1β, transforming growth factor-1 beta; TNF-α, tumour necrosis factor-alpha; VEGF, vascular endothelial growth factor; ↑, increase; ↓, decrease.

## 4. Perspectives

The current review presented evidence on CPC enhancement by several synthetic materials in bone defect healing. The incorporation of various synthetic materials in CPC has been scientifically proven to resolve the limitations of CPC and/or influence the characteristics of CPC in terms of physical, mechanical, and biological properties. The addition of synthetic polymers (such as PLGA and PAAS) resulted in increased setting time, biomechanical strength, and osteogenic properties of CPC. For PLGA, the setting time and compressive strength of the composite were highly dependent on its particle morphology, whereby incorporating dense PLGA leads to proper setting time and increased compressive strength, whereas introducing porous PLGA into CPC prolonged the setting time and decreased compressive strength. Based on the previous evidence, biomimetic materials (such as synthetic collagen and chondroitin sulfate) have been proven to be a potential enhancer for CPC as the reinforced CPC exhibited more superior characteristics of injectability, raised mechanical strength, osteoconductivity, and osteogenic properties than pure CPC, satisfying the requirements for a good bone graft. However, trimethyl chitosan did not improve the compressive strength of the CPC after their combination. Strontium, iron, and zinc reduced, but copper increased the setting time of the incorporated CPC. Most chemical elements and compounds increased biomechanical properties and promoted osteogenic differentiation after addition into CPC. For strontium, both the setting time and compressive strength decreased with increasing content of strontium in the composite. The combination of PLGA with wollastonite or silicon/zinc as an enhancer seemed to increase the flexibility and injectability, which was not observed in the PLGA/CPC composite. The setting time of the composites was also increased when CPC was enhanced by the combination of PLGA and silicon/zinc or alendronate. In addition, alendronate was found to compromise the increased compressive strength conferred by PLGA. The overall characteristics of CPC after the addition of synthetic polymers, biomimetic materials, chemical elements/compounds, or in combination have been summarised (Table 5).

Several limitations of current evidence need to be acknowledged. Young osteoporotic animals have been widely used as a model in most of these studies. The presence of other medical conditions may inhibit bone regeneration and thus should be tested in research using a bone defect model in animals with various types of diseases. In addition, small animals (such as rats, mice, and rabbits) were used as research models for bone defects, which may have different prognoses than large animals such as sheep and bovine. It may take a longer time to exert similar outcomes, understand the mechanisms involved, and validate the mechanisms of the components involved to be translated into clinical settings. The synthetic materials used to enhance CPC summarised in the current review were heterogenous, indicating the lack of original evidence to allow the direct comparison between pure CPC and those enhanced by single or multiple synthetic materials in the same experimental setting.

## 5. Conclusions

In summary, the incorporation of synthetic materials into CPC can help to overcome the drawbacks of the conventional powder-liquid type of cement. The composites displayed enhanced properties in the aspects of mechanical strength and osteogenic activities while retaining CPC’s injectability, mouldability, osteoconductivity, biocompatibility and biodegradability; the various composites possess great potential to be used as materials for implantation in bone defect via minimally invasive surgical techniques. It is recommended to validate the effectiveness of synthetically-enhanced cement in human trials and investigate its effects in more challenging conditions such as the coexistence of infection or disease, poor blood supply, and critical bone defects.

## Figures and Tables

**Table 5:** The characteristics of CPC upon enhancement by different types of synthetic enhancers.

	Polymers	Biomimetic Materials	Chemical Elements/ Compound	Combination of Synthetic Materials
Physical properties	↑ setting time ↓ pore size	↑ injectability ↑ setting time	↑ injectability (iron and copper) ↓ setting time (except copper increased in setting time)	↑ injectability ↑ setting time (except dexamethasone + Laponite^®^ decreased in setting time)
Mechanical properties	↑ compressive strength ↑ flexural strength ↑ work-of-fracture	↑ compressive strength (except trimethyl chitosan, no improvement in compressive strength) ↑ elasticity	↑ compressive strength (except strontium decreased in compressive strength with an increasing percentage of strontium) ↑ flexural strength	↑ compressive strength (except PLGA + alendronate decreased in compressive strength) ↑ flexibility
Biological properties	No cytotoxicity ↑ osteogenesis ↑ bone density ↓ bone erosion ↑ mineralisation ↑ osteogenic differentiation	No cytotoxicity ↑ osteogenesis ↑ bone density (except synthetic collagen I mimetic P-15, no change in bone density) ↑ osteogenic differentiation	No cytotoxicity ↑ osteogenesis ↑ bone density ↑ osteogenic differentiation	No cytotoxicity ↑ osteogenesis ↑ bone density ↑ osteogenic differentiation

Abbreviations: PLGA, poly(lactic-co-glycolic acid); ↑, increase; ↓, decrease.

## Data Availability

Not applicable.
